# Thermally Activated Delayed Fluorescence Organic Dots (TADF Odots) for Time‐Resolved and Confocal Fluorescence Imaging in Living Cells and In Vivo

**DOI:** 10.1002/advs.201600166

**Published:** 2017-01-01

**Authors:** Tingting Li, Dongliang Yang, Liuqing Zhai, Suiliang Wang, Baomin Zhao, Nina Fu, Lianhui Wang, Youtian Tao, Wei Huang

**Affiliations:** ^1^ Key Laboratory for Organic Electronics and Information Displays and Institute of Advanced Materials (IAM)Jiangsu National Synergetic Innovation Center for Advanced Materials (SICAM)Nanjing University of Posts and Telecommunications9 Wenyuan RoadNanjing210023China; ^2^ Key Laboratory of Flexible Electronics (KLOFE) and Institute of Advanced Materials (IAM)Jiangsu National Synergetic Innovation Center for Advanced Materials (SICAM)Nanjing Tech University (Nanjing Tech)30 South Puzhu RoadNanjing211816China

**Keywords:** organic dots (Odots), thermally activated delayed fluorescence (TADF), time‐resolved fluorescence imaging (TRFI)

## Abstract

The fluorophores with long‐lived fluorescent emission are highly desirable for time‐resolved fluorescence imaging (TRFI) in monitoring target fluorescence. By embedding the aggregates of a thermally activated delayed fluorescence (TADF) dye, 2,3,5,6‐tetracarbazole‐4‐cyano‐pyridine (CPy), in distearoyl‐sn‐glycero‐3‐phosphoethanolamine‐poly(ethylene glycol) (DSPE‐PEG2000) matrix, CPy‐based organic dots (CPy‐Odots) with a long fluorescence lifetime of 9.3 μs (in water at ambient condition) and high brightness (with an absolute fluorescence quantum efficiency of 38.3%) are fabricated. CPy‐Odots are employed in time‐resolved and confocal fluorescence imaging in living Hela cells and in vivo. The green emission from the CPy‐Odots is readily differentiated from the cellular autofluorescence background because of their stronger emission intensities and longer lifetimes. Unlike other widely studied DSPE‐PEG2000 encapsulated Odots which are always distributed in cytoplasm, CPy‐Odots are located mainly in plasma membrane. In addition, the application of CPy‐Odots as a bright microangiography agent for TRFI in zebrafish is also demonstrated. Much broader application of CPy‐Odots is also prospected after further surface functionalization. Given its simplicity, high fluorescence intensity, and wide availability of TADF materials, the method can be extended to develop more excellent TADF Odots for accomplishing the challenges in future bioimaging applications.

## 1 Introduction

Fluorescence imaging has been demonstrated as an indispensable and versatile tool in tracking and understanding complex biological environments both in vivo and in vitro.^1^, ^2^ Compared to other imaging modalities, fluorescence imaging offers high spatiotemporal resolution as a noninvasive technique.^1^, ^2^, ^3^ It can probe the deeper layers of a specimen at ambient conditions and also enable spectroscopic diagnosis with chemical sensitivity.^4^ On the other hand, fluorescence imaging suffers from the general limitations, such as high tissue autofluorescence.[[qv: 4a,5,6]] One strategy to separate target fluourescence signals from background autofluorescence relies on the use of fluorophores with emissions at near‐infrared (NIR) wavelengths.[[qv: 6b,7,8]] However, typical organic NIR dyes usually exhibit small Stocks shifts, which can reabsorb emitted photons leading to undesired weak emission and background interferences.^9^ The strategy of fluorescence resonance energy transfer (FRET) was adopted to address this issue.^10^, ^11^ Although effective, such probing systems (i.e., FRET) are structurally more complicated and require more synthetic efforts.

Alternatively, considerable attention has been paid to pursuing suitable fluorescent probes with long‐lived fluorescence lifetime which can be used in time‐resolved fluorescence imaging (TRFI) for eliminating the background fluorescence.[[qv: 4a,5,12]] TRFI can overcome many limitations of fluorescence imaging. For example, introduction of an appropriate delay time between the pulsed excitation light and measurement of the long‐lived luminescence of dyes is expected to eliminate the short‐lived background fluorescence and provide high signal‐to‐noise ratio.[[qv: 13,14a]] Typically, phosphorescent heavy‐metal complexes exhibit relatively long lifetimes because their phosphorescence emits through triplet states with a lifetime of the order of milliseconds.^14^ Several phosphorescent nanoprobes have been reported for optical imaging in vivo, especially for hypoxia bioimaging.^15^ Most of these complexes are based on rare‐earth heavy metals like iridium^15^, ^16^ or europium,^17^ which faces the challenge of potential toxicity.^18^ Although phosphorecence lifetime is long enough to enable the time‐resolved detection, their application in bioimaging is still limited by their drawbacks, such as relatively weak phosphorescence intensity, requirement of sophisticated instrumentation,^15^ and potential damage to surrounding cells and tissues by transferring the energy of their excited triplet state to singlet oxygen.^18^ As a result, there is a demand to develop novel nanoprobes with long‐lived fluorescence, ultrabright emission, and biocompatibility for TRFI applications.

Organic fluorogens exhibiting delayed fluorescence (DF) have drawn intensive interests because they can be used as new generation emitters for organic light‐emitting diodes (OLEDs).^19^ Especially, those emitters exhibit thermally activated delayed fluorescence (TADF) property.^19^, ^20^ The emission mechanism of TADF for OLEDs was first and influentially presented by Adachi and co‐workers.^19^ Typically, TADF originates from charge transfer systems with a thermally accessible gap between the lowest singlet (S_1_) and triplet (T_1_) excited states, which enable efficient reverse intersystem crossing.[[qv: 19a,21]] Obviously, TADF dyes containing no heavy metals have a substantially longer fluorescence lifetime because the population of the excited singlet state originates from the triplet state.[[qv: 5,19a,21]] Since TADF can be realized in molecules with a small overlap between their highest occupied molecular orbital bearing donor units and lowest unoccupied molecular orbital bearing acceptor units, many TADF dyes have been designed and used successfully to improve the light emitting capability of OLEDs.^19^, ^20^ When TADF emitters are used in OLEDs, their long lifetimes always result in the device efficiency roll‐off.[[qv: 19f]] Interestingly, the longer TADF lifetime can be useful for fluorescence lifetime imaging. For example, compound 2′,7′‐dichlorofluorescein‐bis(6‐enyl‐4‐(dicyanomethylene)‐2‐methyl‐4H‐pyran (DCF‐MPYM) based on a fluorescein derivative shows the long‐lived luminescence (22.11 μs in deaerated ethanol) and has been used in time‐resolved fluorescence imaging in living cells.^5^ However, in the in vitro experiments, DCF‐MPYM showed low fluorescence intensity and BSA must be used to eliminate the singlet oxygen to enhance the DF. The sensitivity to singlet oxygen of discrete TADF dye will limit their wide application in TRFI.

Very recently, conjugated polymer dots (Pdots) are emerging as a new class of ultrabright fluorescent nanoprobes for biological imaging.^22^, ^23^, ^24^, ^25^, ^26^ They exhibit several important characteristics for in vitro and in vivo fluorescence studies, such as high brightness, fast emission rate, excellent photostability, nonblinking and nontoxic feature.^23^ Moreover, the dimensions of Pdots can be tuned from several to tens of nanometers without affecting their spectral properties. Fluorescent organic dots (Odots) based on molecular semiconductors with aggregation‐induced emission (AIE) characteristics have also attracted intensive attention,[[qv: 8b,9,27]] because AIE effect provides these organic molecules enhanced luminescence in aggregated states.^28^ Besides, they also possess additional advantages such as easier synthesis and purification, monodispensibility, good biodegradability and chemistry tailorability. For instance, using AIE fluorogens as the fluorescent domain and biocompatible distearoyl‐sn‐glycero‐3‐phosphoethanolamine‐poly(ethylene glycol) (DSPE‐PEG2000) derivatives as the encapsulation matrix, AIE dots in vitro and in vivo cell tracing applications have been successfully explored.^27^ Currently, organic dots and nanoprobes have mainly been used in cell imaging, bio‐/chemosensing, drug and gene delivery, photothermal and photodynamic therapy, photoacoustic imaging, and two‐photon‐excited fluorescence imaging.^29^ However, these applications largely rely on the fluorescence intensity signals instead of their fluorescence lifetime,^23^, ^24^ because DF is still a rare phenomenon to these nanoprobes. Thus, exploration Odots based on TADF dyes is of particular interest as the resulting TADF Odots are expected not only to share all the merits of Pdots and AIE dots but also to have their own long‐lived fluorescence.

**Figure 1:**
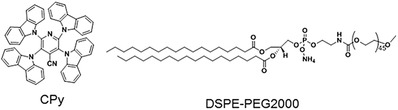
Chemical structures of CPy and DSPE‐PEG2000.

In this regard, we report the preparation of a new CPy‐Odot with bright and long lifetime fluorescence based on a green emissive TADF compound, 2,3,5,6‐tetracarbazole‐4‐cyano‐pyridine[[qv: 20b]] (CPy in this work) (as shown in **Figure** **1**). CPy exhibits enhanced fluorescent emission upon aggregation because it shows higher fluorescent quantum efficiency (QY, 54.9%) in film state than that in its chloroform solution (24.7%).[[qv: 20b]] As measured in degased toluene, CPy exhibits a lifetime of 8.3 μs that is three orders longer than most of the conventional organic dyes with a lifetime ranging from 0.1 to 10 ns.[[qv: 4a]] In present contribution, CPy‐Odots were successfully prepared by us through encapsulating aggregated dots of CPy in DSPE‐PEG2000 matrix.^27^ The prepared CPy‐Odots exhibit several advantages including good water‐solubility, strong brightness (absolute QY of 38.3% in Milli‐Q water), good photostability and biocompatibility. Importantly, CPy‐Odots can emit strong luminescence with a lifetime of 9.3 μs in ambient atmosphere, suggesting great potential for TRFI in vitro and in vivo. The applicability of CPy‐Odots for TRFI was demonstrated in both Hela cells and zebrafish model after assessment of their cytotoxicity. Intense fluorescence signals of CPy‐Odots were detected on the plasma membrane of Hela cells rather than in their cytoplasm or nuclear region. These results also render CPy‐Odots the capability for dynamically tracking plasma membranes or nerve cells.^30^, ^31^

## 2 Results and Discussion

### 2.1 Synthesis of CPy‐Odots

**Figure 2:**
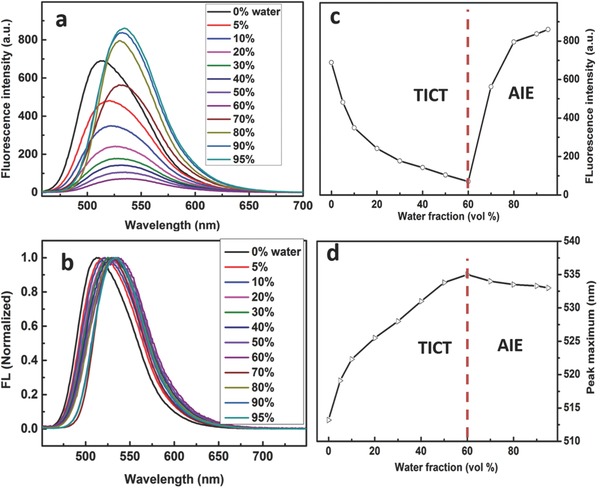
a) PL spectra and b) normalized PL spectra of CPy in mixed solvents of THF/water (water fraction, v/v%) with the final concentration of 9.15 × 10^−6^ m, plot of c) peak intensity and d) peak maximum versus water fraction in the THF–water mixture.

TADF fluorogen of CPy was synthesized according to our previous work. Previously, this compound was applied as a high performance emitter in the OLED devices.[[qv: 20b,c]] In this work, CPy was encapsulated in lipid‐PEG derivative of 1,2‐distearoyl‐sn‐glycero‐3‐phosphoethanolamine‐*N*‐[methoxyl‐(polyethylene glycol)‐2000] (DSPE‐PEG2000)^27^, ^32^ to afford the CPy‐Odots with a long lifetime. Because of the amphiphilic property of DSPE‐PEG2000, CPy aggregates were entangled by the hydrophobic DSPE segments to form the core of CPy‐Odot, whereas the hydrophilic PEG2000 segments extend into the aqueous phase.[[qv: 27a]] Since the Odots' core is formed with aggregated CPy, it is necessary to investigate the photoluminescent properties of CPy in aggregated states before the preparation of CPy‐based Odots. The fluorescence behaviors of CPy in tetrahydrofuran (THF)/water mixture were first investigated.[[qv: 28c,d]] The strong emission of CPy in THF is partially quenched upon its aggregation in the presence of water (**Figure** **2**a,c). Interestingly, the PL intensity exhibits opposite variation at different fractions of water. When the fraction of waster is less than 60% (v/v), higher water content in the THF/water mixture leads to weaker fluorescence emission. In contrast, when the water content reached 70%, the emission enhanced dramatically, even higher than that exhibited in THF solution. This interesting phenomenon can be explained as follows, although no detailed evidence is in hand. At the low fraction of water, CPy, with pyridine unit and strong polar cyanide group, is dissolved in mixed solvents as discrete molecules. At this stage, the emission of CPy aggregates is weakened and is bathochromically shifted from 515 to 534 nm, possibly due to the increase in solvent polarity and hence the transformation to the twisted intramolecular charge transfer state.[[qv: 27c]] After the water fraction is increased to higher than 70%, the hydrophobic effect of CPy results in the formation of CPy aggregates. Thus, the fluorescence emission is coming from the aggregates of CPy, where the aggregation induced emission enhancement (AIEE) dominates the increase of fluorescence intensity. Unlike the conventional AIE compounds, the fluorescence maximum is red‐shifted from 515 nm (0% of water content) to 534 nm (>80% of water content) upon the increase of fraction of water (as shown in Figure 2b,d). The photophysical properties of CPy in adverse solvents with different polarity were also systematically investigated (as shown in Figures S3–S5, Supporting Information). Although the fluorescence intensity of CPy in dimethylsulfoxide (DMSO)/water mixture showed a different trend, the AIEE effect was observed, too. These studies suggest the potential of CPy as a promising AIE fluorophore for fluorescent Odots.^27^, ^28^

**Figure 3:**
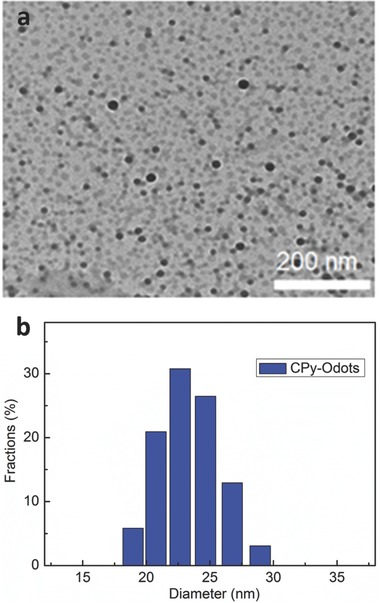
a) TEM image and b) DLS plot of CPy‐Odots.

To prepare CPy‐Odots, DSPE‐PEG2000 was chosen as the encapsulation matrix due to its good encapsulation performance and good biocompatibility.^29^, ^30^ Typically, 1 mL of THF solution containing DSPE‐PEG2000 and CPy with a constant weight ratio of 2:1 was first diluted with 2 mL of THF/water (5/1, v/v) injected into 7 mL of Milli‐Q water, followed by sonication and THF evaporation under stirring (Scheme S1, Supporting Information). The pellucid CPy‐Odots suspension was obtained after passing through a 0.22 μm syringe filter and then stored at 4 °C for further use. CPy‐Odots obtained after freezing‐dry can be homogeneously redispersed in Milli‐Q water or photphate buffer saline (PBS) buffer solution. Transmission electron microscopy (TEM) images revealed that CPy‐Odots were spherical in shape with the diameter at 17 nm level (**Figure** **3**). The average size of the CPy‐Odots in water suspensions was 23 ± 5 nm determined by dynamic light scattering (DLS) measurements. The slightly smaller size measured from TEM was attributed to shrinkage of CPy‐Odot in dry state, which is a common phenomenon for organic NPs.^24^, ^27^

### 2.2 Photophysical Properties of CPy‐Odots

**Figure 4:**
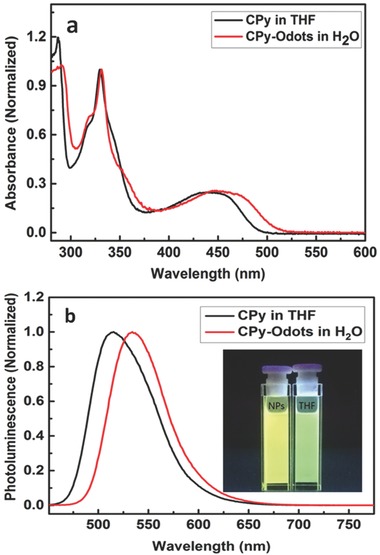
a) UV–vis absorption and b) PL spectra of CPy in THF (black) and CPy‐Odots in Milli‐Q water (red), respectively (inserted picture: fluorescence of CPy and CPy‐Odots under 365 nm excitation).

**Figure 5:**
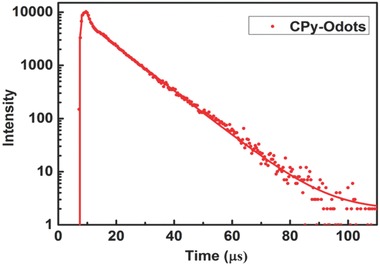
Fluorescence emission lifetime of CPy‐Odots in Milli‐Q water at ambient atmosphere (300 K in air, excited at 405 nm and fluorescence collected at 520 nm).

The photophysical properties of CPy and CPy‐Odots were measured using UV–vis absorption and PL spectroscopy (**Figure** **4**). According to the UV–vis absorption spectra for CPy in THF with the concentration of 1.83 × 10^−5^ m, the strong bands at 285 and 335 nm are attributed to the absorption of carbazole units and its conjugated backbone. The longer wavelength broad absorption between 400 and 500 nm for CPy is ascribed to the intramolecular charge transfer (ICT) transition between the electron‐donating carbazole and electron‐withdrawing cyanopyridine. The weak ICT absorption band indicates the restriction of charge transfer from the carbazole donor parts to cyano‐pyridine part, which is the requirement for TADF materials designation.^19^ Compared to that of CPy in THF solution, the ICT absorption band of CPy‐Odots in water showed slight red‐shift, indicating strong molecular aggregation induced by π–π stacking. CPy is virtually strong luminescent when molecularly dissolved in good organic solvents such as THF, CH_2_Cl_2_, CHCl_3_, toluene, etc. As illustrated in Figure 4b, CPy in THF solution exhibits a strong fluorescent emission with a maximum at 515 nm, and the measured fluorescence QY is around 24%. The as‐prepared CPy‐Odots in Milli‐Q water exhibit a higher fluorescence QY of 38.3%.[[qv: 21a]] The QY enhancement is achieved by isolating CPy aggregates by DSPE‐PEG2000 encapsulating layer from the nonsolvent of water. The PL decay of CPy‐Odots was measured by a time‐correlated single photon counting technique at room temperature in air. The decay profile is fitted well with a biexponential decay as shown in **Figure** **5**. The lifetime (τ) is dominated by a long decay component of 9.4 μs plus a small contribution from the short decay of 2.3 ns. The weighted‐average lifetime is ≈9.3 μs. The lifetime of CPy‐Odots prepared by different conditions also provides nearly the same lifetime, such as in PBS buffer (Table S1, Supporting Information). These results indicate the long lifetime and intense fluorescence emission of CPy can be maintained when the Odots were prepared and used as fluorescent nanoprobe in various buffer solutions.

**Figure 6:**
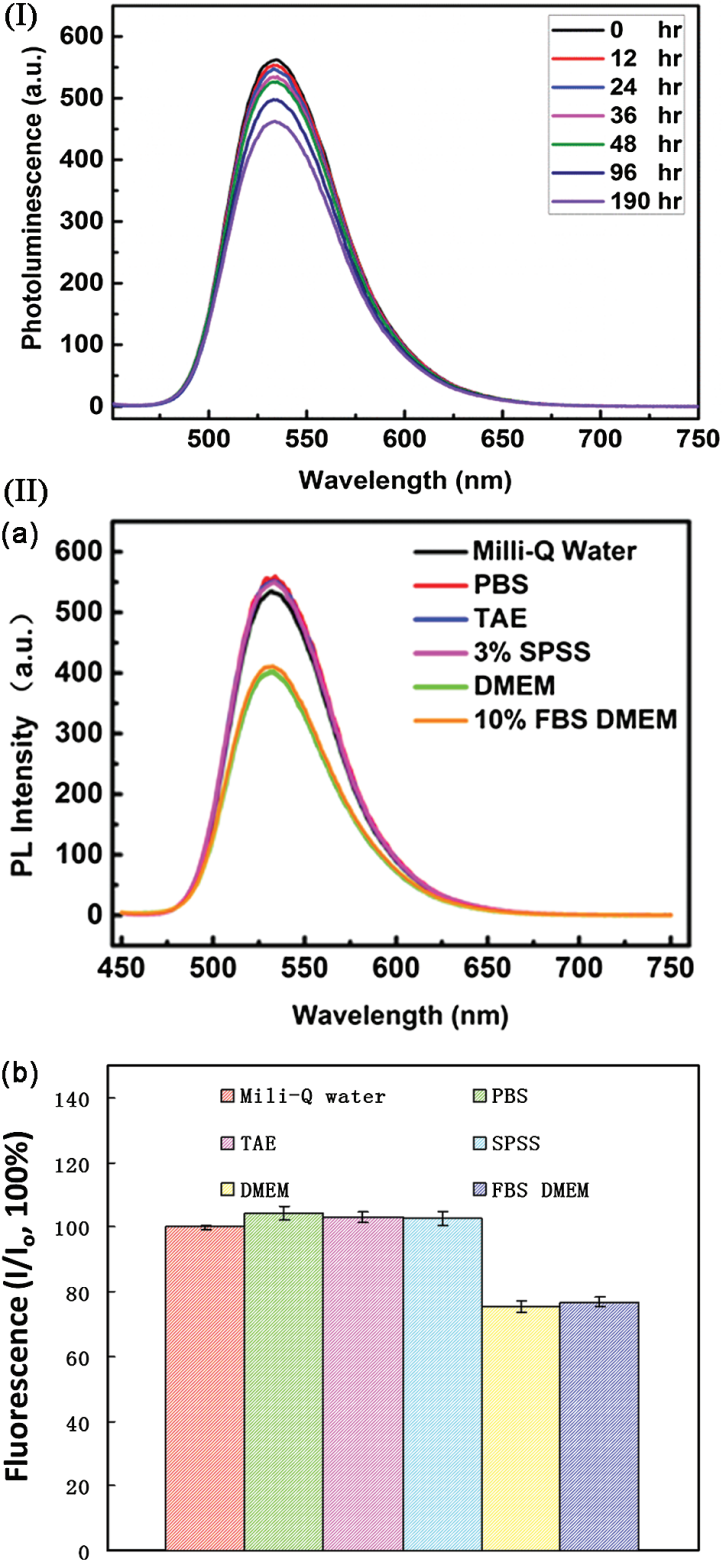
(I) Fluorescence spectra of 6.10 × 10^−5^ m CPy‐Odots in Milli‐Q water under ambient condition, measured after freeze‐drying CPy‐Odots redispersed in Milli‐Q water for 0, 12, 24, 36, 48, 96, and 190 h (data collected without vigorous shaking or undergoing ultrasonication). (II) a)Fluorescence intensity of dried CPy‐Odots redispersed in Milli‐Q water, PBS, TAE, 3% SPSS, DMEM, 10% FBS‐DMEM. b)The Milli‐Q water solution was used as control for the initial fluorescence intensity (*I*_0_) and *I* is the fluorescence intensity of each sample, Fluorescence(%) was calculated by *I*/*I*_0_ ([CPy‐Odots] = 6.10 × 10^−5^ m).

We measured the intensity of fluorescence emission from CPy‐Odots under adverse conditions since the emission efficiency of Odots is critically important for many fluorescence‐based biological applications. Besides, it is also a sensitive way to monitor nanoparticle stability.[[qv: 25f]] According to our investigation, the resulting CPy‐Odots exhibited excellent photostability under different conditions. The intense fluorescence emission of drop‐casting film of CPy‐Odots were maintained more than 90% fluorescence intensity of as‐prepared film when exposed to 405 nm laser (irradiation power of 55.6 mW cm^−2^) for 2800 s (Figure S6, Supporting Information). Specifically, the fluorescent intensity of Cpy‐Odots was still stable after continuous exposure to visible light at ambient condition for more than 190 h (**Figure** **6** **–I**). Moreover, Figure 6‐II shows the fluorescence intensities of as‐prepared CPy‐Odots in Milli‐Q water and CPy‐Odots redispersed in PBS, tris‐acetate‐EDTA (TAE) buffer solution, and physiological conditions (i.e., Dulbecco's modified Eagle medium (DMEM)). In comparison to the fluorescence intensity of the Milli‐Q water solution, the fluorescence was not or slightly quenched in PBS, TAE, 3% SPSS solution and only decreased by 20% in DMEM and fetal bovine serum (FBS) containing DMEM. Fortunately, all of these solutions showed excellent fluorescence stability after they are stored at 37 °C for 2 d, with no compromise of fluorescent lifetime (remains around 9 μs). These results proved that CPy‐Odots are photostable and stable enough for use in biological environment without interference by the buffer solutions.

### 2.3 Confocal and Fluorescence Lifetime Imaging of Living Cells

**Figure 7:**
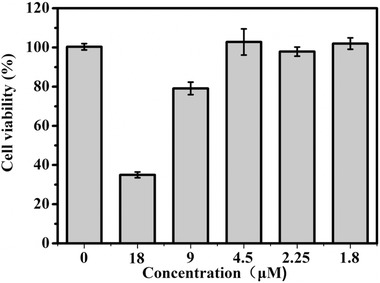
Cell viability of CPy‐Odots in Hela cell line at various concentrations after 24 h of incubation. Mean value and standard deviations were obtained from four independent experimental determinations.

Since our Odots have excellent PL properties and good stability, we further investigated their potential use as fluorescent nanoprobes for fluorescence imaging in living cells. Cytotoxicity of the CPy‐Odots after 24 h of incubation with human cervical cancer cells (Hela cells) were first evaluated using 3‐(4,5‐dimethyl‐2‐thiazolyl)‐2,5‐diphenyltetrazolium bromide (MTT) assays. The result shows that the cell viability remains above 80% after a 24 h incubation with 9 × 10^−6^ m Cpy‐Odots (calculated to be 7.1 mg mL^−1^), as illustrated in **Figure** **7**. This result indicates low cytotoxicity and good biocompatibility of CPy‐Odots during the bio‐test period.

**Figure 8:**
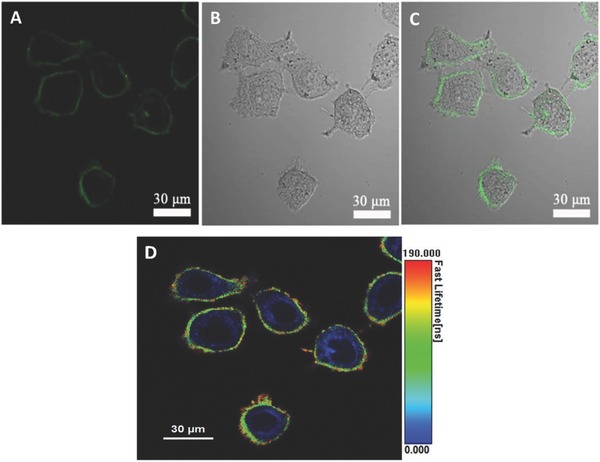
Confocal fluorescence images of HeLa cells after incubation solely with 0.5 × 10^−6^ m CPy‐Odots in PBS buffer solution for 2 h. A) Confocal image recorded under excitation at 405 nm with 480–580 nm bandpass filters. B) Bright field image. C) Corresponding overlayed image. D) Fluorescence lifetime images.

The confocal and fluorescence lifetime imaging were conducted on Hela cells after incubation with CPy‐Odots (0.5 × 10^−6^ m) in PBS for 2 h. Staining HeLa cells with CPy‐Odots led to strong PL emission from the cell membrane rather than in the cytoplasm of Hela cells (**Figure** **8**A). The intensive green emission originated from CPy‐Odots can be observed clearly. To confirm the photostability of CPy‐Odots in cellular environment, the fluorescence images of Hela cells, after they were incubated with CPy‐Odots, were collected. The stained Hela cells were irradiated with 405 nm laser for 30 s per 10 min, then the fluorescence images were captured. As shown in Figure S7 (Supporting Information), intense fluorescence emission from CPy‐Odots in cellular membrane still can be observed after 12 h operation. This study suggests the great potential of CPy‐Odots for long‐term tracking due to its good photostability. The fluorescence lifetime images of Hela cell are shown in Figure 8D. The observed blue color at cytoplasm is mainly ascribed to the background short‐lived fluorescence lifetime signals from cell cytoplasm, whereas the yellow to red color from the cell periphery is owing to long‐lived fluorescence lifetime signals of CPy‐Odots. The fluorescence lifetime imaging results also indicate the emission signals from CPy‐Odots on the cell membrane with an average lifetime of 165 ns. Thus, on the basis of long‐lived fluorescence signals, the autofluorescence can be removed and the signal‐to‐noise ratio can be improved significantly.

**Figure 9:**
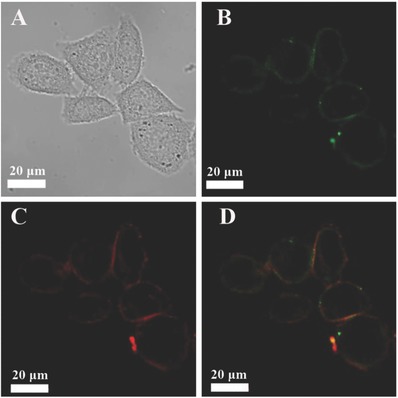
Fluorescence images of HeLa cells costaining with CPy‐Odots and DiIC_18_, simultaneously: A) Bright field. B) Confocal fluorescence image collected with 480–580 nm bandpass filters for CPy‐Odots upon excitation at 405 nm. C) Confocal fluorescence image collected with 580–680 nm bandpass filters for DiIC_18_ upon excitation at 559 nm. D) Corresponding overlayed image of (B) and (C).

**Figure 10:**
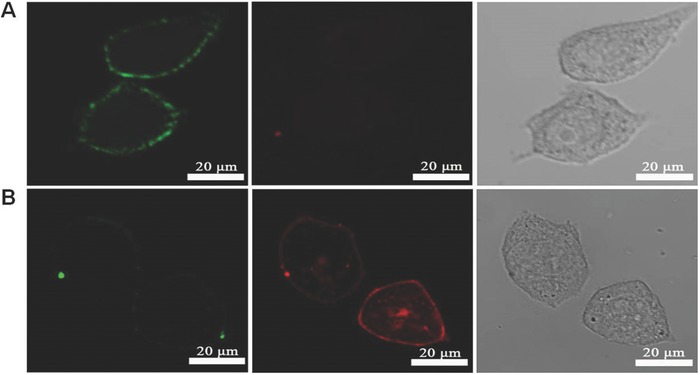
Confocal fluorescence images of HeLa cells incubated with CPy‐Odots (at a dots concentration of 0.5 × 10^−6^ m for 1 h) and DiI (5 × 10^−6^ m for 15 min) sequentially, A channel (Sample I) stained with CPy‐Odots first, B channel (Sample II): stained with DiIC_18_ first. Left column, confocal fluorescence images recorded with 480–580 nm bandpass filters for CPy‐Odots upon excitation at 405 nm. Middle column, confocal fluorescence images recorded with 580–680 nm bandpass filters for DiIC_18_ upon excitation at 559 nm. Right column, corresponding overlayed images.

**Figure 11:**
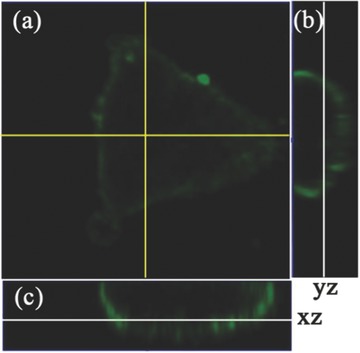
Z‐scan images of living Hela cell incubated with CPy‐Odots: a) an *xy* image obtained at *z* = 0.7 μm, b,c) the *yz* and *xz* cross‐sections (*z* = 0–19 μm) taken at the lines shown in panel (a), respectively.

Since no bioconjugation was performed on CPy‐Odots, this kind of specific membrane labeling property seems very interesting. To confirm the CPy‐Odots were localized on the plasma membranes, Hela cells were costained with CPy‐Odots and 1,1′‐dioctadecyl‐3,3,3′,3′‐tetramethylindocarbocyanine perchlorate (DiIC_18_), a typical cell membrane marker.^5^, ^30^ **Figure** **9**A was the fluorescence image collected at 480–580 nm for CPy‐Odots labeled Hela cells upon excitation at 405 nm and Figure 9B was collected at 580–680 nm for DiIC_18_ upon excitation at 559 nm. The overlap of green‐ and red‐fluorescence emission confirms the hypothesis that CPy‐Odots can specifically localize on cell membranes like DiIC_18_. Additionally, a sequential staining method was also used to confirm this membrane labeling behavior. For example, sample I was prepared by incubating Hela cells with CPy‐Odots for 1 h then with DiIC_18_ for another 15 min, whereas sample II was prepared by incubating Hela cells first with DiIC_18_ for 15 min then with CPy‐Odots for another 1 h. Both samples were recorded by confocal fluorescence microscopy. As shown in **Figure** **10**A, cells first stained with CPY‐Odots (green) were unable to be stained by DiIC_18_ anymore (red). In contrast, when cells were first stained by DiIC_18_, significant red fluorescence emerged with minimal green signal generated from CPy‐Odots (Figure 10B). Note that, DiIC_18_ has defused into the cytoplasm (red emission in the cytoplasma region) through common endocytosis pathway.[[qv: 30b]] According to the results of sequential staining experiments, it can be concluded that CPy‐Odots and DiIC_18_ are located at the same position of Hela cells. It should be mentioned that CPy‐Odots can be stably retained in the plasma membrane even after incubation with cells for more than 2 h, which is an advantage over DiIC_18_ (DiIC_18_ tends to label entire cells via lateral diffusion in few minutes).^31^ Furthermore, this kind of membrane probing is also supported by Z‐scan image of the CPy‐Odots stained cells, as shown in **Figure** **11**. Since no customized bioconjugation was performed on CPy‐Odots, a plausible interpretation of observed results is as follows. The shell‐layer of DSPE‐PEG2000 containing long‐aliphatic chains is highly lipophilic. When the cellular uptake process takes place, the CPy‐Odots will interact with the cell membrane. The phospholipids in the immediate neighborhood of the site of contact are drawn to the surface of the Odots, leading to membrane wrapping and encapsulation.^27^, ^32^ During this stage, on one hand, the weak interaction between DSPE and the phospholipids layer of cell membrane leads the Odots to retain in the membrane. On the other hand, the lipophilic CPy dye leaves from the nanoparticle of CPy‐Odots, then inserts into the cell membrane. Considering that the average of fluorescence lifetime observed by TRFI measurements, which is much shorter than the values obtained from the aqueous solution of CPy‐Odots, the CPy‐leaving‐from mechanism seems more plausible, because the fluorescence lifetime of TADF dyes were always very sensitive to the singlet oxygen. Besides, the fluorescence lifetime of CPy in different conditions were measured and summarized in Table S2 (Supporting Information). In addition, the small sizes of our Odots make them have an enhanced ratio of surface area to volume, which thermodynamically increases the binding energy between Odots and the phospholipids layer. Similar explanations on the effect of particle size on membrane wrapping have been reported previously.^33^ Hence, the interaction of Odots with cellular membrane renders CPy‐Odots the membrane probing activity.

### 2.4 In Vivo Fluorescence Imaging

**Figure 12:**
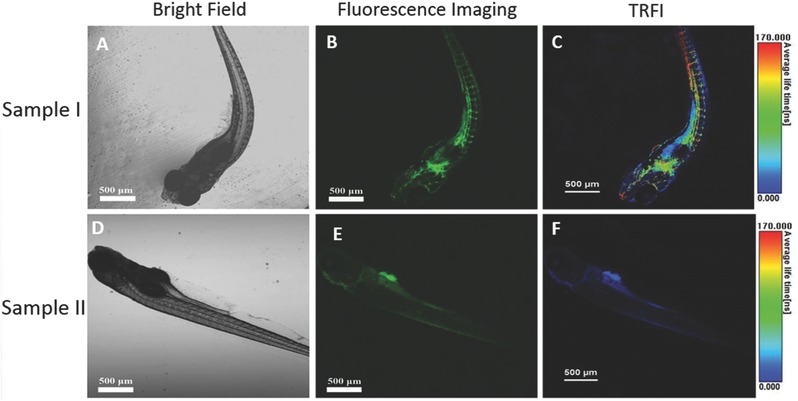
Confocal fluorescence images of zebrafish: A–) zebrafish injected with CPy‐Odots; D–F) zebrafish noninjected with CPy‐Odots; (A, D) were bright field images; (B, E) were confocal fluorescence images recorded with 480–580 nm bandpass filters for CPy‐Odots upon excitation at 405 nm; (C, F) were lifetime images.

To explore the potential of CPy‐Odot as an in vivo imaging probe, we applied confocal and time‐resolved fluorescence imaging measurements in living zebrafish.^34^ Briefly, the zebrafish was still alive after being injected with 300 nL CPy‐Odots into the ventricle. As shown in **Figure** **12**B, the vascular network of zebrafish could be visualized clearly from the fluorescence image. The weak fluorescence of other organs was originated from background emission (see in Figure 12E). Obviously, it was difficult to identify the fluorescence of CPy‐Odots from the background signals (Figure 12B,D). However, by comparing the images captured with fluorescence lifetime imaging microscopy (Figure 12C,F), we can easily distinguish the vivid green to red signals of CPy‐Odots with longer fluorescent lifetime from the bioluminescence with the lifetime shorter than 3 ns under excitation at 405 nm. This study indicates CPy‐Odots can be used as bright microangiography agents for FLIM in living zebrafish.

**Figure 13:**
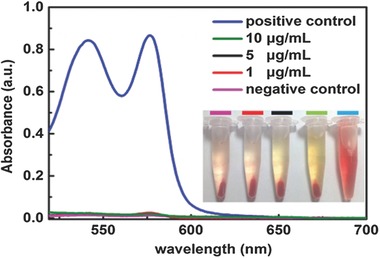
Hemolysis assay for CPy‐Odots, using Milli‐Q water and PBS as positive control and negative control, respectively. The CPy‐Odots were at 1, 5, and 10 μg mL^−1^.

Since CPy‐Odot shows the potential as the bright angiography agent, it is necessary to evaluate the hemolytic behaviors of CPy‐Odots on Zebrafish red blood cells (RBCs).^35^ RBCs were isolated from the whole blood by centrifugation and purified by five times washing with physiological saline.[[qv: 35a]] 30 μL of packed erythrocytes was resuspended in 1.2 mL physiological saline with various concentrations of CPy‐Odots. Pure water and PBS were selected as positive control and positive control, respectively. After incubating these samples for 2 h at 37 °C, the samples were spun down for the detection of hemoglobin released from hemolyzed RBCs. The hemoglobin content in supernatant was measured using a UV–vis spectroscopy at 540 nm (see in **Figure** **13**). A good hemocompatibility (<2% hemolysis) of CPy‐Odots is confirmed at concentrations as high as 10 μg mL^−1^. The results provide the evidence that CPy‐Odots are hemocompatible nanoprobes for in vivo fluorescence imaging and other applications.

## 3 Conclusion

In summary, the current study provided the facile approach toward long lifetime fluorescent nanoprobes based on a TADF dye. The nanoprobes of CPy‐Odots were successfully fabricated through DSPE‐PEG2000 matrix encapsulation method. The small‐sized, stable, bright, and biocompatible CPy‐Odots with long lifetime fluorescence emission (≈9.3 μs in ambient atmosphere in various buffer solutions) hold great potential as nanoprobes in the time‐resolved and confocal fluorescence imaging in living cells and zebrafish. An unexpected cellular membrane specific labeling behavior of CPy‐Odots was observed, which was further confirmed by control experiments using DiIC_18_. Attractively, our CPy‐Odots exhibited the superior membrane retention than DiIC_18_. Additionally, we have demonstrated the application of CPy‐Odots as bright microangiography agents for in vivo fluorescence imaging in living zebrafish. To the best of our knowledge, this is the first report on developing long lifetime fluorescence nanoprobes by facile embedding the aggregates of organic TADF dyes into diblock polymer matrix. Considering the wide availability of TADF dyes used in OLEDs, the nanotechnology which stemmed from our studies can spread its expandability to other TADF dyes for establishment of multipurpose TRFI nanoprobes. By encapsulating various components into the CPy‐Odots, the applications of TADF‐based long lifetime Odots will be easily broadened in future integrated detections and therapeutic systems. We expect our strategy of synthesis of TADF dye‐based nanoprobes can open new opportunities for TRFI in future biological and medical research and clinic applications.

## 4 Experimental Section

*Materials*: Carbazole–pyridine hybrid TADF material, 2,3,5,6‐tetracarbazole‐4‐cyano‐pyridine (CPy) was synthesized according to the previous work.[[qv: 20a,b]] 1,2‐Distearoyl‐sn‐glycero‐3‐phosphoethanolamine‐*N*‐[methoxy(polyethylene glycol)‐2000] (DSPE‐PEG20002000) was purchased from Shanghai Seebio Biotech, Inc. DMEM, FBS, 3‐(4,5‐dimethylthiazol‐2‐yl)‐2,5‐diphenyl tetrazolium bromide (MTT), and 1,1′‐dioctadecyl‐3,3,3′,3′‐tetramethylindocarbocyanine perchlorate (DiIC_18_) were obtained from Invitrogen. The antibiotic agents penicillin–streptomycin (100 U mL^−1^) were purchased from Life Technologies. THF was provided by Aladdin reagent (Shanghai, China). Milli‐Q water used in the experiments was made from a Milli‐Q Plus System (Millipore Corporation, Bedford, MA, USA). Hela cells were obtained from KeyGEN Biotechnology Company Ltd (Nanjing, China). All pH measurements were conducted with a Eutech PH 700 pH‐meter. Unless otherwise noted, all chemicals were obtained from commercial suppliers and used as received.

*Preparation of Odots*: The CPy‐Odots in aqueous solution were prepared via a well‐documented matrix‐encapsulation method with optimization. DSPE‐PEG2000 (Scheme S1, Supporting Information) was chosen as the encapsulation matrix due to its good encapsulation performance and high biocompatibility.^27^, ^32^ In a typical preparation, CPy (1 mg) and DSPE‐PEG2000 (2 mg) were dissolved in 1 mL THF by stirring for 3–4 h under inert atmosphere. Then, the homogeneous solution was further diluted using a mixture of THF and Milli‐Q water (2 mL, water volume fraction of 20%). After this diluted solution was sonicated for 2 min, it was added quickly to 7 mL of Milli‐Q water in a bath sonicator. THF was removed by N_2_ stripping before the solution was concentrated to 5 mL by rotary evaporator. Finally, the formed CPy‐Odots suspension was filtered through a 0.22 μm syringe filter. The obtained CPY‐Odot solution was with a concentration of 0.2 mg mL^−1^. The clear solution was stable and could be stored at 4 °C for more than four months without signs of aggregation.

*Characterization Method*: The particle size and morphology of the CPy‐Odots were characterized by DLS and TEM. DLS was performed using a 1 cm disposable polystyrene cuvette at 25 °C. Average particle sizes were determined by laser light scattering with a particle size analyzer (90 Plus, Brookhaven Instruments Co., Holtsville, NY, USA) at a fixed angle of 90° at room temperature. Samples for TEM measurements were prepared by drop casting the Odots dispersion onto copper grids. The samples were allowed to dry at room temperature, then the TEM images were obtained using a Hitachi H‐H7500 microscope operated at 120 kV. UV–vis absorption spectra were recorded with Shimadzu UV‐3600 spectrophotometer. Fluorescence spectra were recorded by using a RF‐5301PC spectrofluorophotometer. Excited‐state lifetime studies were performed with an Edinburgh LFS‐920 spectrometer with a hydrogen filled excitation source. The data were analyzed by iterative convolution of the luminescence decay profile with the instrument response function using a software package provided by Edinburgh Instruments. The absolute quantum yields of the complexes were determined through an absolute method by employing an integrating sphere.

*Cytotoxicity by MTT Assay*: The Hela cells were cultured in RPMI 1640 medium supplemented with 10% FBS at 37 °C in an incubator containing 5% CO_2_. Cells (1 × 10^5^ mL^−1^) were placed on a 15 mm diameter glass‐bottomed culture dish and allowed to adhere for 24 h before fluorescence images were captured.

The viability of Hela cells was evaluated with methylthiazolyldiphenyltetrazolium bromide (MTT) method. In brief, the cells were seeded on each well of 96 wells plate. The CPy‐Odots at concentrations of 18, 9, 4.5, 2.25, 1.8 × 10^−6^ m were added to the wells of the experiment group. After incubation at 37 °C, 5% CO_2_ for 24 h, the original medium in each well was removed completely. Subsequently, 180 μL of DMEM (without FBS) and 20 μL of MTT stock solution (5 mg mL^−1^ in PBS) were added before 4 h incubation. Then, the plate was incubated for 3 h at 37 °C. The formazan dye reduced by live cells succinodehydrogenase was dissolved with 150 μL DMSO. The absorbance of each well at 490 nm was recorded using a microplate spectrophotometer (PowerWave XS2, BIOTEK, US). Then the cell viability was calculated by the mean absorbance value of experiment group divided by mean absorbance value of control multiply by 100. Five replicates were run for each concentration.

*Hemolysis Studies*: Zebrafish blood was collected as described by Murtha et al.[[qv: 35a]] The whole blood was centrifuged and washed five times with physiological saline. 30 μL of packed erythrocytes was resuspended in 1.2 mL physiological saline containing different concentrations of CPy‐Odots and pure water. Then, the erythrocytes were incubated at 37 °C for 2 h and were centrifuged at 2000 rpm for 5 min. Finally, the hemoglobin content in supernatant was measured using a UV–vis spectroscopy at 540 nm. The hemolysis activity was calculated by (sample absorbance − negative control absorbance)/(positive control absorbance − negative control absorbance) × 100%.[[qv: 35b]]

*In Vitro Cell Imaging*: Hela cells were precultured in 6‐well plates containing cell culture coverslips to achieve 80% confluence. The medium was then removed and the cells were washed twice with PBS buffer. HeLa cells were incubated with CPy‐Odots and fresh RPMI 1640 medium mixture (final concentration 0.5 × 10^−6^ m) at 37 °C for 2 h. The cells were then washed with PBS buffer solution twice to remove nonspecifically bound CPy‐Odots. The fluorescent images were recorded on confocal microscope (Olympus, FV1000, Japan) under excitation at 405 nm (1 mW). The fluorescence signal was collected at 480–580 nm. Fluorescence lifetime images were taken by confocal microscope with fluorescence lifetime imaging upgrade kit (PicoQuant) using a 405 nm picoseconds pulsed diode laser (PDL 800‐D) at 2.5 MHz repetition rate.

In order to verify CPy‐Odots were stained on the cell membrane, Hela cells were incubated with CPy‐Odots first for 1 h and with DiI (5 × 10^−6^ m) for 15 min at 37 °C (sequential costaining experiment), then HeLa cells were irradiated using 405 and 559 nm lasers equipped with scanning confocal microscopy, and the collected fluorescence channels were 480–580 and 580–650 nm, respectively.

*In Vivo Fluorescence Imaging*: Zebrafish as animal model was purchased from Nanjing YSY Biotechnology Company Ltd. 300 nL CPy‐Odots (0.1 mg mL^−1^) was injected into ventricle using a reported procedure.^34^ The Zebrafish was anesthetized in 7% diethyl ether and transferred into 1.5% carboxymethyl cellulose sodium. Then, fluorescence image and lifetime images of CPy‐Odots‐injected zebrafish were recorded using the same instrument as described in cell imaging part.

## Supporting information

As a service to our authors and readers, this journal provides supporting information supplied by the authors. Such materials are peer reviewed and may be re‐organized for online delivery, but are not copy‐edited or typeset. Technical support issues arising from supporting information (other than missing files) should be addressed to the authors.

Supplementary
