# Supplementary material for: Thermally Activated Delayed Fluorescence Organic Dots (TADF Odots) for Time‐Resolved and Confocal Fluorescence Imaging in Living Cells and In Vivo
Source: Adv Sci (Weinh). 2017 Jan 1;4(4):1600166. doi: 10.1002/advs.201600166 (PMC5396166; doi:10.1002/advs.201600166)
Supplement: Supplementary file 1 — Supplementary [file ADVS-4-na-s001.pdf]

Copyright WILEY-VCH Verlag GmbH & Co. KGaA, 69469 Weinheim, Germany, 2016.

ADVANCED SCIENCE Open Access

Supporting Information

for *Adv. Sci.,* DOI: 10.1002/advs.201600166

Thermally Activated Delayed Fluorescence Organic Dots (TADF Odots) for Time-Resolved and Confocal Fluorescence Imaging in Living Cells and In Vivo

Tingting Li, Dongliang Yang, Liuqing Zhai, Suiliang Wang, Baomin Zhao, Nina Fu, Lianhui Wang,* Youtian Tao, and Wei Huang**

Copyright WILEY-VCH Verlag GmbH & Co. KGaA, 69469 Weinheim, Germany, 2013.

Supporting Information

Thermally Activated Delayed Fluorescence Organic Dots (TADF Odots) for Time-Resolved and Confocal Fluorescence Imaging in Living Cells and in Vivo

Tingting Li, Dongliang Yang, Liuqing Zhai, Suiliang Wang, Baomin Zhao, Nina Fu, Lianhui Wang,* Youtian Tao, Wei Huang**

Materials and Instruments

All reagents and chemicals were procured from commercial sources and used without further purification. The NMR spectra were recorded on a Bruker spectrometer at 400 MHz with $CDCl_3$ as the solvent. All chemical shifts are reported in the standard δ notation of parts per million (ppm). Fluorescence spectra were recorded by using a RF-5301PC spectrofluorophotometer**.** Excited-state lifetime studies were performed with an Edinburgh LFS-920 spectrometer with a hydrogenfilled excitation source. The data were analyzed by iterative convolution of the luminescence decay profile with the instrument response function using a software package provided by Edinburgh Instruments. The absolute quantum yields of the complexes were determined through an absolute method by employing an integrating sphere.

Experimental Section

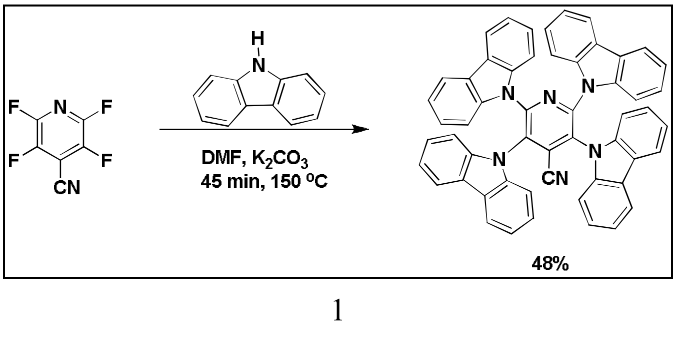

Synthesis of CPy: In a 100 mL Schlenck tube, carbazole (2.05 g, 12 mmol), 2,3,5,6-fl uoro-4-cyano-pyridine (350 mg, 2.0 mmol) and K_2CO_3 (4.15 g, 35 mmol) were mixed in anhydrous DMF (30 mL). After the mixture was heated at 150 °C for 45 min, the reaction was quenched by addition of water (50 mL). Then, CPy was extracted with chloroform. The organic layer was further washed with water and brine for twice. Pure CPy was isolated by silica column chromatography with the eluent of hexane/dichloromethane (4/1 to 1/2). Pure CPy is a yellowish solid with a yield of 48%. ^{1}H NMR (400 MHz, $CDCl_3$) δ (ppm) 7.82 (m, 4H), 7.76 (d, J = 8.1 Hz, 4H), 7.46 (d, J = 8.1 Hz, 4H), 7.29 (m, 4H), 7.15 (m, 8H), 7.10 (t, 4H), 7.04 (t, 4H) ^{13}C NMR (100 MHz, $CDCl_3$) δ (ppm) 147.60, 138.58 138.47, 130.26, 125.95, 124.43, 121.63, 120.52, 119.99, 111.93, 110.50, 109.74.

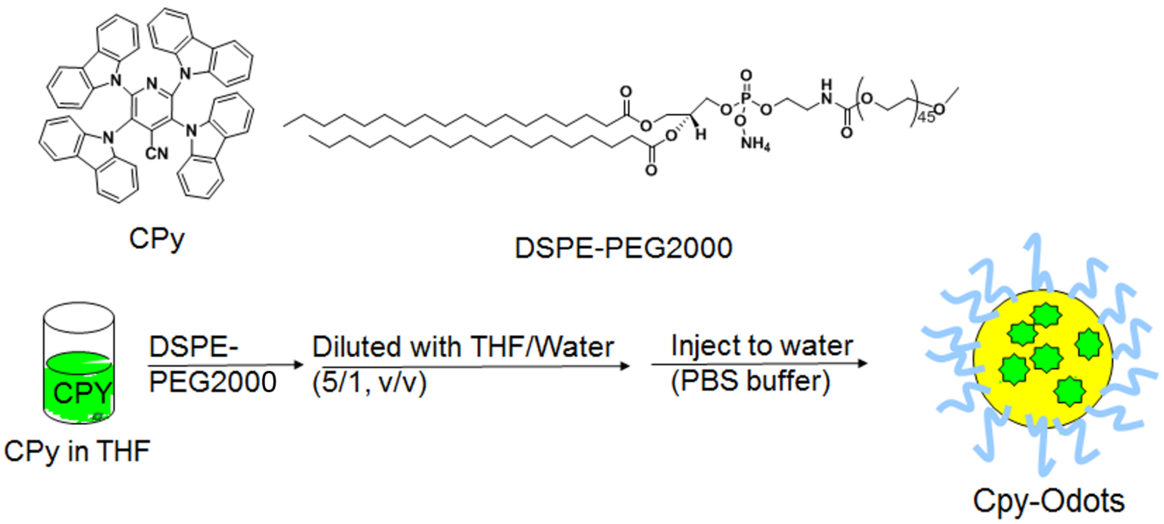

Scheme S1. Chemical structure of CPy and DSPE-PEG2000 and the Scheme illustration of synthesis of TADF Odots.

Table S1. Fluorescence quantum efficiencies and fluorescence lifetimes measured with various samples obtained by reproducing of CPy-Odots.

Sample entry [a]	Fluorescence Quantum Efficiency[b]	Fluorescence Lifetime τ (μs)
1	0.44	9.4
2	0.38.[illegible]	9.6
3	0.32	9.2
4	0.35	9.6
5	0.39	10.5
PBS-1	0.41	9.8
PBS-2	0.26	9.6

Table notes: a) Sample 1–5: prepared by injection 1 mL of THF solution containing CPy and DSPE-PEG2000 to Milli-Q water, sample PBS-1 and PBS-2: prepared by injection 1 mL of THF solution containing CPy and DSPE-PEG2000 to PBS buffer solution; b) determined through an absolute method by employing an integrating sphere.

Table S2. Fluorescence lifetimes of CPy measured with various conditions.

Sample entry	Fluorescence Lifetime τ (μs) [a]
In air saturated toluene	4.9 (ns)
In well degassed toluene	8.3
CPy aggregates [b]	3.4
CPy-Odots	9.3

Table notes: a) all of these data were measured under ambient conditions (300 K, in air); b) CPy aggregates prepared by the addition of non-solvent of water to the THF solution of CPy.

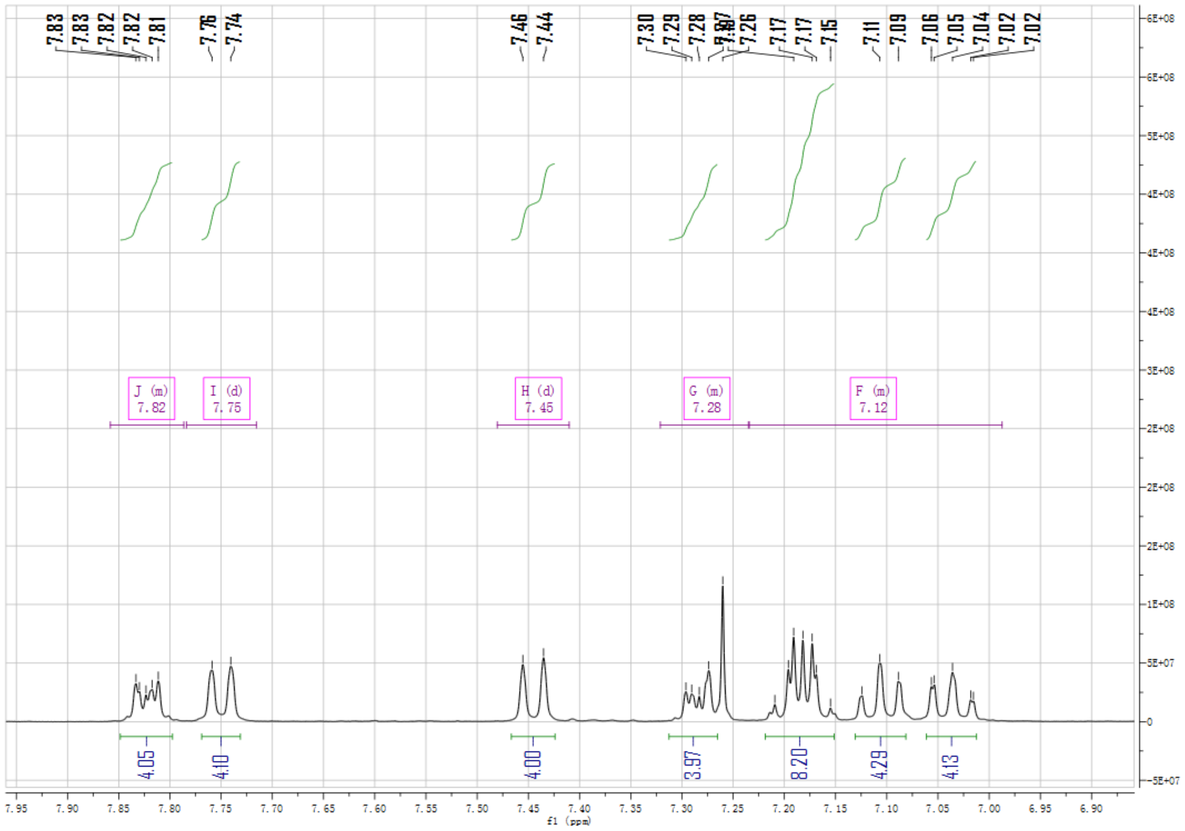

Figure S1. ^{1}H NMR spectrum of CPy in $CDCl_3$.

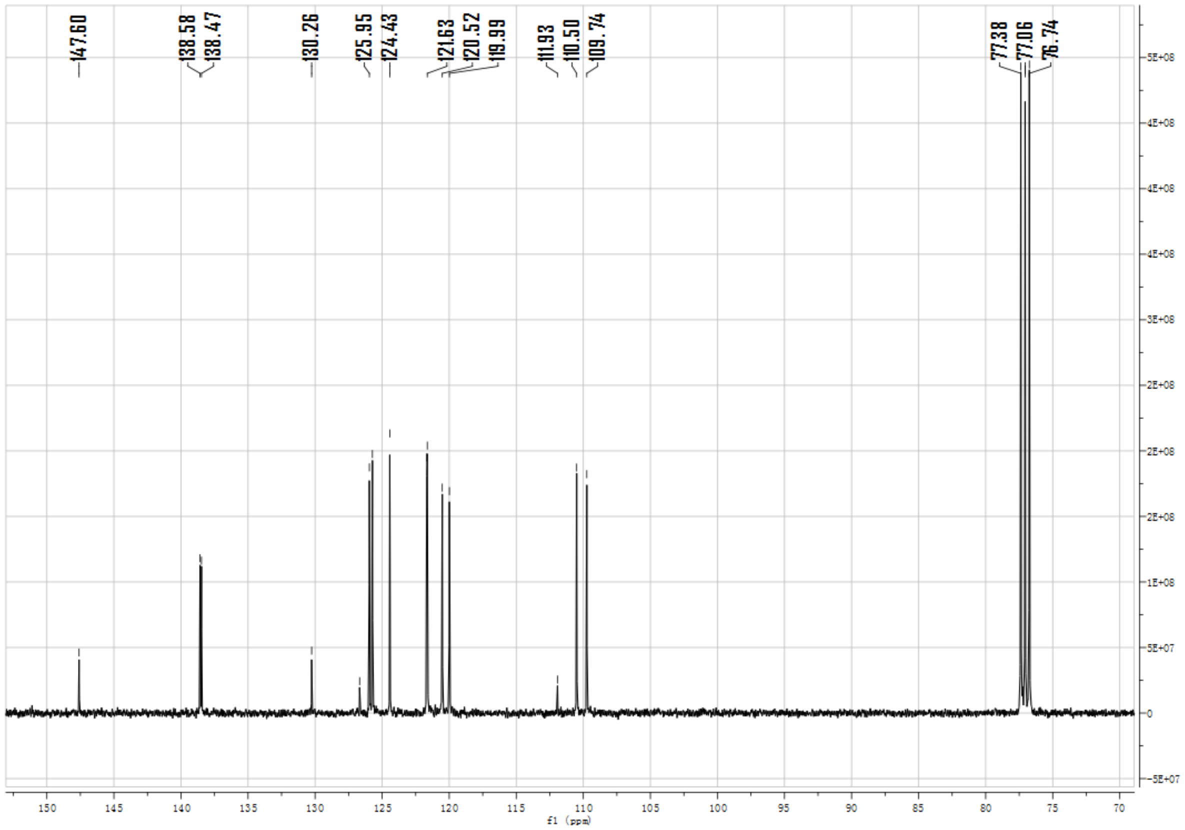

Figure S2. ^{13}C NMR spectrum of CPy in $CDCl_3$.

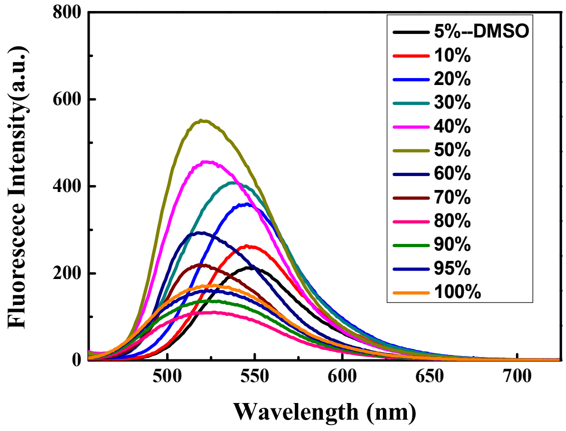

Figure S3. PL spectra of CPy in mixed solvents of DMSO/water (DMSO fraction, v/v%) with the concentration of CPy is 1.31×10^{-5} M.

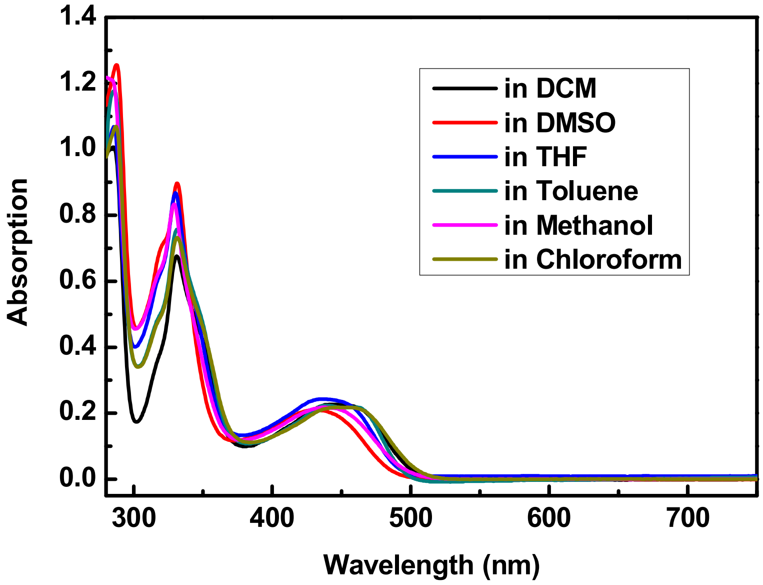

Figure S4. UV-vis absorption spetra of CPy in adverse solvents of DCM (black), DMSO (red), THF(blue), Toluene (green), Methanol (purple) and Chloroform (brown). The concentration is 1.31 × 10−5 M.

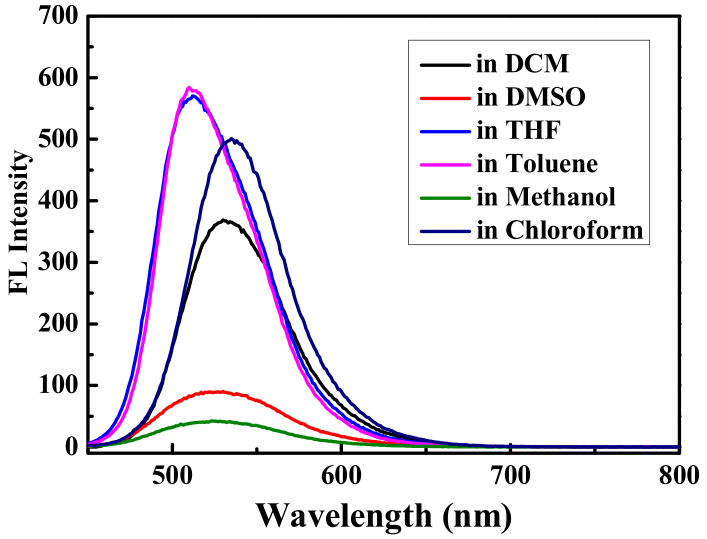

Figure S5. Fluorescence spetra of CPy in adverse solvents of DCM (black), DMSO (red), THF(blue), Toluene (green), Methanol (purple) and Chloroform (brown). The concentration is 2.62×10^{-6} M.

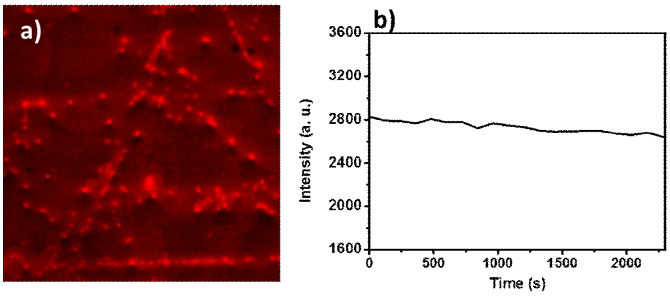

Figure S6. a) Fluorescence image of drop-casting film of CPy-Odots, b) fluorescence intensity of the drop-casting film of CPy-Odots (excitation power was 55.6 mw/cm^2 and excitation wavelength was 405 nm)

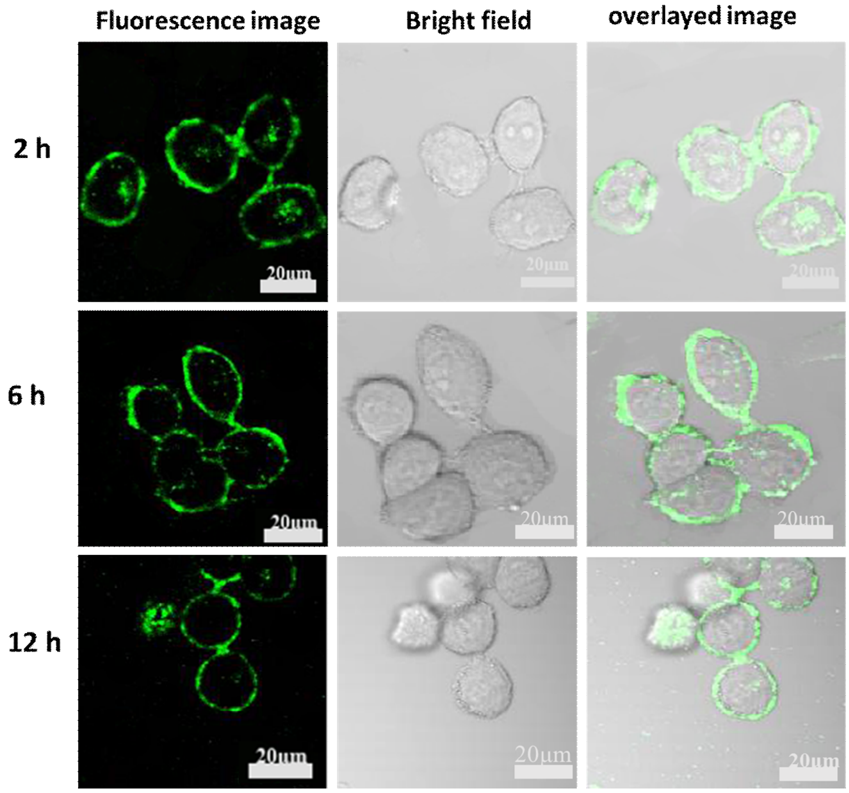

Figure S7. Fluorescence images of Hela cells after incubation with CPy-Odots and exposed to 405 nm laser for 30 second per10 min. 2h line) after the cell was kept for 2 h; 6h) after the cell was kept for 6 h; 12 h line) after the cell was kept for 12 h. Fluorescence image collected at 480~580 nm for CPy-Odots upon excitation at 405 nm. The concentration of CPy-Odots used was 1.5 μM.
